# Notes and News

**Published:** 1889-10-26

**Authors:** 


### Notes and News.

COLLECTED AND COMMUNICATED.

In another column will be found a full statement of the events which have taken place at the City-road Chest Hospital in connection with the dismissal of the secretary, which should be read by all who take an interest in hospitals. In the face of these facts, quite apart from many other matters, which we understand will have to be brought to the notice of the governors if the proposed meeting is ever held, we cannot conceive it possible that many, if any, governors will be found to vote for a resolution censuring the council for its action in the matter.

The opening meeting of the present session of the Hospitals Association was held on Wednesday evening, October 16, in the board-room of Charing Cross Hospital. Dr. Bristowe, F.R.C.P., F.R.S., president of the society, took the chair at 8 p.m. There was a considerable attendance, those present representing the various interests concerned in hospital work and management. The President read a paper on the work of the Hospitals Association, reviewing its progress from its inception in 1883 down to the present time. Sir Wm. Moore suggested that those desiring information upon various points should ask questions, which would be answered by the president or some member of the council. Mr. Burdett said the president had not referred to a matter engaging the attention of the Government at the present time—namely, the question whether or not there shall be a Royal Commission to enquire into our voluntary system. This association had remained neutral on that question, holding that, whereas there was no possible objection to enquiry, still an enquiry was not then necessary for the prosperity and development of the hospitals. What was really required at the present time for the voluntary medical charities of the metropolis was a uniform system of accounts; a plan to prevent the multiplication of hospitals in any locality, whether a hospital were needed there or not; and a movement which would ensure that every penny which was subscribed by the public should be brought properly to account, and shown in the public statements of the institution to which it was given under its proper head. These matters could be secured in a Bill of about two clauses, which would not take much time in passing through the House of Commons. It would be desirable to go a step further, and provide that a building opened for private reasons should not be recognised as a public hospital without proper enquiry and full justification. As to the ambulance branch of the society's work, he might say that the placing of street ambulances within distances of some 400 yards throughout the four-mile radius, was progressing steadily and satisfactorily. They had had a competition of ambulance litters, and had determined to try two, and within the next few weeks they would place the Hospitals Association litters in many parts of London. At the present time a man might be knocked down in the street and suffer from a common fracture, but owing to the want of proper appliances, and the manner in which the man was conveyed to hospital, his injury might be turned into a most serious one, which, if not ending fatally, might maim him for life. He believed their ambulance work would take rank with anything that society had accomplished in the six years of its existence.

Dr. Hentsch urged the association to use its influence in inducing hospital authorities to use the funds and the institutions committed to their care for the exclusive use of the necessitous sick. He did not think the hospitals were founded for paying patients; but patients were now received in the hospitals who could well afford to pay a general practitioner. He spoke in the interest of the general practitioner, and did not care about anyone else. He thought also hospital authorities should not take it on themselves to send away patients to provident dispensaries, which were ruining the profession and pauperising the public. The authorities should not dictate to patients where they should go. There were plenty of general practitioners about, some of them almost starving. Besides, in these dispensaries doctors were placed under the thumb of a committee—middle men—a system which was very degrading to the profession. But he was glad to see that some engaged in the consulting branch of the profession were now feeling, at the City-road Chest Hospital, what it was to be placed under middle men.

Dr. Steele, medical superintendent of Guy's Hospital, said there was no medical provision for the poor when Guy's was founded 200 years ago. Now dispensaries and infirmaries were provided for the poor all over the kingdom, and were more numerous than the voluntary medical charities. It was not paupers, but the industrious poor, who had the benefit of hospitals. Other patients were recommended by medical men, who said they were able to pay a certain amount—from 5s. to £1 1s. a week for a bed. At Guy's, within the last few years, they had reduced the number of out-patients from some 70,000 to about 30,000 a year. He proposed a vote of thanks to the president for his interesting address and for presiding at this meeting.

Dr. Gilbart Smith, in seconding this, said that, as a member of the council of the Metropolitan Provident Dispensary Association, which had been attacked by Dr. Hentsch, he could say that the scheme for enabling the working classes to avail themselves of the benefit of that association was drawn up by general practitioners from all parts of London. He (Dr. Smith) was not aware that patients, sent away from any hospital because of their ability to pay, were sent to provident dispensaries. In respect to the City-road Chest Hospital, the matter stood thus: A paid official having had serious charges brought against him, for which he was severely censured, chose in his defence to bring forward a charge that the medical staff were guilty of recklessly experimenting on patients, not for the patients' good, but for their own. That charge, if found true, should undoubtedly lead to the dismissal of the medical staff. But the charge was investigated, not at the request of the secretary, but at the request of the medical staff. It was found false, the secretary was dismissed, and he now chose to plead that this grave charge was a matter of privilege, and as such should not have been made public. He was championed by men who, while they praised the staff and deplored the act of the secretary, yet proposed that the staff should be dismissed, and the secretary retained—an illogical position, which showed the exact condition of affairs. In a few weeks the public would know the true state of the case. Meanwhile the council had no anxiety as to the result.

Dr. Bristowe made a few remarks on the discussion, agreeing with Mr. Burdett that a Bill of a few clauses dealing with hospital accounts would be a benefit.

The president announced that the next general meeting of the association would be held on November 20, when Sir William Moore, K.C.I.E., late Surgeon-General Bombay Staff, would read a paper on "Leprosy and Leper Houses."

The following vacancies are announced this week, the amount of the salary in each case being given after the name of the institution:

**Resident Medical Officers.**

Weston-super-Mare Hospital. House Surgeon. — £50, board, &c.
Middlesbrough Infirmary. House Surgeon.
Carlisle Counties Asylum. Junior Assistant Medical Officer. £80, board, &c.
Liverpool Eye and Ear Infirmary. House Surgeon.—£80, board, &c.
North-West London Hospital. Two Clinical Assistants.
Liverpool Dispensaries. Two Assistant Surgeons.—£80, board, &c.
Salop Infirmary. House Surgeon.—£100, board, &c.
Flintshire Dispensary. House Surgeon.—£100, rooms, &c.
Wolverhampton General Hospital. House Surgeon.—£100, board, &c.

**Non-Resident Medical Officers.**

Birmingham General Hospital. Assistant Physician.—£100.
Wittesley Parish. Medical Officer—£70.
University College, Liverpool. Lecturer on Children's Diseases.
Liverpool Royal Infirmary. Honorary Surgeon.
Victoria Hospital for Sick Children. Ophthalmic Surgeon.
Charing Cross Hospital. Surgical Registrar.

The authorities of the St. Raphael's Home for the consumptive poor, Worthing, desire us to state that during the six months of its existence ninety-five patients have been received. Amongst these not a single death has occurred, many have derived benefit, and in some incipient cases the disease has been arrested, and the patients returned to the duties of life. A special appeal for donations towards the increased winter expenses of firing and lighting has just been issued by the hon. sec., Mrs. St. A. Horton.

The aptness with which a madman will turn an argument is well known. The following is an amusing instance. An inspector visiting the asylum at X. was requested by the medical superintendent to be very careful to address a certain patient as "your imperial majesty," the poor man imagining himself to be Julius Cæsar, and becoming furious if he did not receive what he considered proper respect. The inspector was careful to follow instructions, and all went well. On a subsequent visit he again addressed the patient by the same title. "What do you mean?" was the reply; "don't talk nonsense. I'm Plato." "Oh!" said the inspector; "I beg your pardon, but I thought you were Julius Cæsar last year." "Well, yes," replied the lunatic; "so I was, but that was by another mother!"

The popularity of medicine as a science is exemplified by the *St. James's Gazette*, which constantly has articles on different drugs, or on surgical operations. Last week it gave a translation from the *Archives de Médecine* on some results of using cocaine. Dr. Baratoux mentions the case of a druggist, who, under the impression that he was attacked by diphtheria, sprayed his throat with a solution of cocaine; for seven or eight hours he passed from one syncope to another, until he finally succumbed. Dr. Abadie reports another case—a woman of seventy-one. She received a hypodermic injection of four centigrammes of cocaine in her lower eyelid before undergoing a trivial operation in that region. At the close of the operation she fainted, and her face became purple as in asphyxia. In spite of the fact that artificial respiration was performed, and that hypodermic injections of ether and caffeine were made, and though the latter seemed for a moment to revive her, this unfortunate woman died five hours afterwards. Mr. St. Clair Buxton, of the Western Ophthalmic Hospital, wrote pointing out that these cases might alarm nervous persons, whereas when cocaine was merely applied to the surface, as oculists use it, it is perfectly safe.

About 10 infants are suffocated weekly in London, yet few are the mothers who are taught how delicate is the breathing apparatus of their babies. Excess of zeal is often the cause of suffocation. A mother wraps her infant too tightly up in a shawl to carry it through the streets, and on reaching her destination is surprised to find her burden dead. The proper way to treat infants ought to be taught in every girls' school.

The Invalid Transport Corps of the St. John Ambulance Association is steadily growing, yet has room for many more members. The good work done by the corps on such occasions as Lord Mayor's Day, and the Trafalgar riots, shows what a useful organisation it is. The subscription is 10s. a year, and once efficient, members need only attend twenty drills in each year. The uniform is very becoming, so there ought to be no lack of recruits.

The harvest festival at the Hospital for Sick Children last Saturday was a prettier and more pathetic sight than can be seen elsewhere in London. The exquisite little chapel was richly decorated with luxuriant flowers, ferns, and fruit, and lit with numerous wax candles. On one side of the aisle were the nurses in uniform, each in charge of some tiny patient; on the other side were numerous visitors. Round the door were gathered the students and porters, and they all joined heartily with their strong voices in the simple children's hymns. After service tea was served in the wards, and the visitors were shown round and made to take an interest in the hospital and its work. Everything is wonderfully dainty and bright at the Hospital for Sick Children, Great Ormond-street, just now, and the wards are well worth a visit from those interested in hospital work.

At the invitation of the head master, Miss Philippa Hicks lately visited University School, Hastings, and gave the boys an account of the Hospital for Sick Children from its earliest days down to the present date. Miss Hicks told them about the twofold difficulty of having to maintain the present building (free of debt), while the chief energy had to be expended on the building of the new wing. The result was the 120 boys promised to maintain a cot, or, in other words, subscribe annually £40. They even made their first collection towards it there and then, which amounted to £3, and they collected quite a large parcel of books, games, puzzles, etc., for the patients. In fact, the whole-hearted way in which they took the matter up seemed such a generous thing on the part of a number of strong, healthy boys, who can have little or no conception of what weakness and ill-health is, that it thoroughly deserves to be put on record.

The interviewer has been attacking Mr. Ernest Hart in his character of President of the Smoke Abatement Institute. Mr. Hart thinks black fogs might be easily done away with, though mists London would always be subject to, being on clay. What is wanted is an Act which shall invest the local authorities of every district with the power of enforcing the construction of smoke-consuming grates in all new buildings, for by far the largest amount of smoke is now made by private houses and in our suburbs. A new city is built up around London nearly every year, and, instead of making these new cities smokeless, they are permitted to add to the impurity of the atmosphere by no restrictions being placed on the builders in regard to the grates in the houses. We are obliged to drain our houses properly lest we pollute the water supply, and we are obliged to build party-walls a certain thickness. How can it be more unjust to enforce the proper consumption of coal and smoke lest we pollute the air we breathe?

